# Copper, Iron, Selenium and Lipo-Glycemic Dysmetabolism in Alzheimer’s Disease

**DOI:** 10.3390/ijms22179461

**Published:** 2021-08-31

**Authors:** Jan Aaseth, Anatoly V. Skalny, Per M. Roos, Jan Alexander, Michael Aschner, Alexey A. Tinkov

**Affiliations:** 1 Department of Research, Innlandet Hospital Trust, P.O. Box 104, N-2381 Brumunddal, Norway; jaol-aas@online.no; 2 World-Class Research Center “Digital Biodesign and Personalized Healthcare”, IM Sechenov First Moscow State Medical University (Sechenov University), Bolshaya Pirogovskaya St., 2–4, 119146 Moscow, Russia; skalny3@gmail.com; 3 Department of Bioelementology, K.G. Razumovsky Moscow State University of Technologies and Management, Zemlyanoi Val St., 73, 109004 Moscow, Russia; 4 Institute of Environmental Medicine, Karolinska Institute, 171 77 Stockholm, Sweden; per.roos@ki.se; 5 Norwegian Institute of Public Health, P.O. Box 222 Skøyen, 0213 Oslo, Norway; jan.alexander@fhi.no; 6 Department of Molecular Pharmacology, Albert Einstein College of Medicine, 1300 Morris Park Avenue, Bronx, NY 10461, USA; michael.aschner@einsteinmed.org; 7 Laboratory of Molecular Dietetics, IM Sechenov First Moscow State Medical University (Sechenov University), Bolshaya Pirogovskaya St., 2–4, 119146 Moscow, Russia; 8 Laboratory of Ecobiomonitoring and Quality Control, Yaroslavl State University, Sovetskaya Str. 14, 150000 Yaroslavl, Russia

**Keywords:** copper, iron, glycemic dysregulation, GLP-1 agonist, advanced glycation end-products, selenium

## Abstract

The aim of the present review is to discuss traditional hypotheses on the etiopathogenesis of Alzheimer’s disease (AD), as well as the role of metabolic-syndrome-related mechanisms in AD development with a special focus on advanced glycation end-products (AGEs) and their role in metal-induced neurodegeneration in AD. Persistent hyperglycemia along with oxidative stress results in increased protein glycation and formation of AGEs. The latter were shown to possess a wide spectrum of neurotoxic effects including increased Aβ generation and aggregation. In addition, AGE binding to receptor for AGE (RAGE) induces a variety of pathways contributing to neuroinflammation. The existing data also demonstrate that AGE toxicity seems to mediate the involvement of copper (Cu) and potentially other metals in AD pathogenesis. Specifically, Cu promotes AGE formation, AGE-Aβ cross-linking and up-regulation of RAGE expression. Moreover, Aβ glycation was shown to increase prooxidant effects of Cu through Fenton chemistry. Given the role of AGE and RAGE, as well as metal toxicity in AD pathogenesis, it is proposed that metal chelation and/or incretins may slow down oxidative damage. In addition, selenium (Se) compounds seem to attenuate the intracellular toxicity of the deranged tau and Aβ, as well as inhibiting AGE accumulation and metal-induced neurotoxicity.

## 1. Introduction

Alzheimer’s disease (AD) is a neurodegenerative condition of high prevalence in older age. In developed countries almost 10% of people over the age of 65 suffer from AD [1] and AD prevalence is increasing globally. During the upcoming decades AD will contribute to an increased socio-economic burden and severe suffering for patients and caregivers. Against this background, measures to modify the serious course of AD are urgently needed.

Most AD patients suffer from the sporadic form, the genetic AD accounting for less than 2% of diagnosed cases [2]. Many sporadic AD patients (almost 25%) are carriers of the ApoE4 allele of apolipoprotein E (chromosome 19) [3]. The mechanisms by which the ApoE4 allele contributes to AD and to increased levels of amyloid beta (Aβ) are unknown, yet the same allele appears to be related to telomere shortening and accelerated aging [4]. It is known that the import of cholesterol into the neurons is achieved by apolipoprotein E (ApoE) via receptors low-density lipoprotein receptor-related protein1 and 8 (LRP1 and LRP8) on the cell surface. It has been suggested that a co-causal factor for AD is changed receptor function of LRP1 or LRP8, and that an underlying cause is inappropriate neuronal supply of cholesterol combined with dysmetabolism of the copper chaperone APP (amyloid precursor protein) [5]. In particular, the essential trace element selenium (Se) is also transported as selenoprotein P (SELENOP) to the brain via a low-density lipoprotein receptor, viz. the LRP8 receptor [6]. In addition, another lipoprotein receptor (LRP1) is implicated in the clearance of Aβ from the brain to the peripheral circulation [7]. Interestingly, this clearance appears to be dependent on the ApoE isoform, as carriers of the apoE4 allele exert reduced efflux of Aβ from the brain, as assessed in in vitro models [8]. Combined with aging hypertension, dyslipidemia and metabolic syndrome (MetS) are risk factors for developing sporadic AD [9].

Language problems and declining recall of recent events are early AD characteristics [10]. These symptoms are attributed to dysfunctions in the hippocampal area of the temporal lobe [11]. Degenerations develop gradually in both the temporal and parietal regions of the brain. These changes are accompanied by a deterioration of hippocampal cholinergic signaling [12]. In clinical medicine, acetylcholinesterase inhibitors have been adopted to alleviate synaptic dysfunction in AD, acting by increasing acetylcholine levels at synaptic loci, however without arresting the disease progression [13]. Symptomatic relief is also achieved by memantine, a compound that acts by blocking the functional dominance of the transmitter N-methyl-aspartate [14]. However, transmitter dysfunctions observed in AD patients are not considered the initiating events in the pathogenesis. Biochemical events initiating AD development most probably reside in some basic impairments of vital intracellular proteins, e.g., the microtubule-associated proteins responsible for the neuronal integrity.

Co-occurrence and comorbidity of AD with other human disorders often occur due to a misbalance of glucose metabolism and other metabolic dysregulations [15]. Pathogenetic mechanisms common to AD and MetS have been described [16,17]. Patients with MetS show an increased risk for vascular complications and oxidative stress, accompanied by inflammation and metabolic dysregulation [18].

The aim of the present review is to discuss traditional hypotheses on the etiopathogenesis of AD as well as the role of metabolic-syndrome-related mechanisms in AD development, with a special focus on advanced glycation end-products and their role in metal-induced neurodegeneration in AD.

## 2. On Traditional Hypotheses for AD Etiology

### 2.1. Physiological APP Processing and Functions of Aβ

The amyloid precursor protein (APP) is a glycosylated protein that is uniformly found in cell membranes, most abundantly in the brain. It has been presumed that the membrane-bound protein APP acts as a copper chaperone, thereby exerting cytoprotective functions [19]. In addition, APP is involved in neuronal system development through participation in synaptic formation and functioning, promotion of axonal growth and formation of neuromuscular junctions [20]. The APP molecule is degraded to several peptides by the three intracellular enzymes: α-, β- and γ-secretases. Soluble cleavage products might also have cytoprotective effects, e.g., on synaptic structures. Specifically, α-secretase-derived secreted amyloid precursor protein (APPsα) possesses neuroprotective and neurotrophic activity [21]. Another secreted APP ectodomain variant APPsβ was also shown to possess biological activity through regulation of certain metabolic pathways [22], although its neuroprotective effect is less pronounced as compared to APPsα [23].

At the same time, not only secreted APP ectodomain variant products possess physiological effects. Although being a pathogenetic molecular basis of AD at increased levels, extremely low Aβ levels were also shown to play a physiological role in the nervous system. Specifically, it is proposed that picomolar levels of Aβ may promote neurite outgrowth and neuroprotection, and even possess antibacterial effects [24]. Similarly, beneficial effects of the tau protein in the maintenance of microtubule integrity and functioning, axonal growth and synaptogenesis were demonstrated [25]. However, these beneficial neurotropic effects are observed only upon physiological control over amyloidogenesis and physiologically low concentrations of Aβ and tau, whereas dysregulation of this process results in a shift to Alzheimer’s disease.

### 2.2. The Amyloid Cascade Hypothesis and the Immunotherapeutic Concept

However, in AD, a less soluble variant, the Aβ-peptide with 42 amino acids, usually referred to as Aβ, is formed in excess and makes up the amyloid core in the characteristic precipitated plaques [26,27].

Based on this typical trait of AD, vaccination with the complete Aβ (1–42) or smaller fragments has been evaluated in transgenic mouse models. Early human tests using the complete Aβ molecule for vaccination resulted in serious adverse events including aseptic meningoencephalitis [28]. Later vaccines have made use of shorter Aβ fragments and some of these vaccines have reached the clinical phases of development, showing antibody response in treated patients without serious adverse reactions, but without significant therapeutic effect [29]. Another immunological option is passive immunization with administration of antibodies against Aβ, which has reduced cerebral amyloid load in transgenic animals. Some monoclonal antibodies against Aβ fragments have also been tried out clinically, but without significant clinical improvement in humans [30].

Pyroglutamate-modified Aβ peptides that are formed upon glutaminyl cyclase catalysis are strongly associated with AD [31]. Therefore, antibodies to pyroglutamate-modified Aβ peptides as well as glutaminyl cyclase inhibitors may be considered as an additional immunotherapeutic approach to AD [31].

### 2.3. New Insights into the Intracellular Metabolism of Aβ

The endoplasmic reticulum (ER) is considered the site of synthesis for all non-degraded APP, while only a minor fraction of Aβ might be secreted from ER [32]. A main part of the Aβ appears to be formed in the neuronal cytosol by abnormal degradation of APP. The last step in an abnormal cleavage of APP leads to formation of Aβ in addition to another fragment named APP Intra-Cellular Domain (AICD), with the latter fragment apparently also exerting cytotoxic actions. Physiologically, AICD and also Aβ can, for the most part, be degraded through the ubiquitin-proteasome system [33]. As for Aβ, it has been reported that it can lead to formation of pathologic aggregates after its appearance in the cytosol [34], these aggregates showing similarities with perinuclear aggresomes [35]. Insufficient activity of the ubiquitin-proteasome system occurring in elderly individuals may lead to enhanced accumulation of insoluble cytosolic aggregates. An effective ubiquitin-proteasome system is the cytosolic prerequisite for selective degradation of different forms of damaged proteins [36]. In healthy young subjects, this machinery can rapidly and selectively degrade moderately damaged or oxidized cell proteins [37]. Recently, it has been found that the activity of the proteasome is reduced during aging, as the proteases are increasingly inhibited by elevated contents of oxidized and cross-linked protein aggregates [38]. Furthermore, it has been observed that the activity of the proteasome is decreased in AD brains compared to age-matched controls, which has been attributed to overloading of precipitates with deranged tau [39]. It is clear that tau degradation by the proteasome is in part ubiquitin-dependent [40]. Interestingly, intracellular Aβ-oligomers can inhibit proteasome activity [41]. These observations indicate that the ubiquitin-proteasome machinery is deranged in AD, leading to the pathologic accumulation of Aβ, together with deranged tau and oxidized proteins inside the nerve cells. It has been reported from a study in a rodent model that modification of the cysteine residues in proteasomes, by addition of a thiol-reactive chemical, reduced their function [42], confirming a previous presumption that cysteinyl group oxidation may be one of the mechanisms for the loss of proteasome activity in elderly individuals [43]. Physiologically, repair of oxidised protein thiol groups is carried out by the actions of reduced glutaredoxin and reduced thioredoxin. Glutaredoxin uses the cysteine-containing tripeptide glutathione (GSH) as a reducing cofactor, whereas the regeneration of thioredoxin to its active form depends on the activity of the selenoenzyme thioredoxin reductase. Optimized intakes of essential sulfur amino acids and of selenium to fortify the selenoenzymes have been recommended in a previous paper [44].

The ubiquitin-proteasome system in neurons appears to be overloaded and deranged in AD, ultimately leading to cell death. Oxidized proteins contribute to a pathologic overloading in aging. Cell deterioration and death lead to extracellular escape of Aβ deposits. Age-related mitochondrial dysfunctions may accelerate the formation of protein oxidation products [45].

Accumulation of damaged mitochondria is common in brain tissues from AD patients and in AD animal models, in addition to autophagosomes, which seem to be formed at mitochondrial endoplasmic reticulum contact sites (MERCS) [46] together with Aβ plaques [47]. It has been hypothesized that formation of abnormal MERCS in AD might lead to mitochondrial dysfunction due to an influx of calcium into mitochondria from the endoplasmic reticulum and Aβ aggregates blocking mitochondrial export of Ca ^++^, in addition to dysfunctional autophagosome synthesis [48]. Physiologically, the major pathway for removal of damaged mitochondria is the ATP-dependent ubiquitin proteasome pathway in addition to mitophagy. A tempting hypothesis is that, together with the overloaded proteasome pathway, a defective mitophagy may play a role in the AD etiopathogenesis [49].

### 2.4. Tau Hyperphosphorylation in AD

Tau is a neuronal, microtubule-associated protein crucial for the function of microtubules in healthy brains [50]. Physiological phosphorylation regulates tau protein binding to microtubules. Under healthy conditions the tau protein remains soluble and adequately phosphorylated. However, dysfunction of the ApoE receptors LRP1 and LRP8 may represent causal factors for the observed microtubule derangement by disrupting the supply of essential nutrients such as cholesterol and selenium. Apparently, tau hyperphosphorylation occurring in AD compromises its normal functions and leads to formation of insoluble neurofibrillary tangles forming bundles of protein filaments [51]. Phosphorylating kinases and de-phosphorylating phosphatases in tandem regulate this process. Increased expression of active kinases has been described in AD, one of these kinases being cyclin-dependent kinase 5 (CDK5) [52]. Inhibitors of CDK5 show neuroprotective properties in in vitro and in vivo AD models [53].

## 3. Hypothesis Involving Metabolic Syndrome and Glycation

### 3.1. The Hypothesis of a Role of Metabolic Syndrome and Dyslipidemia

Clinical studies indicate that metabolic syndrome with dyslipidemia, hypertension, obesity and insulin resistance are significant risk factors for the development of AD [54]. Insulin resistance with elevated levels of advanced glycation end-products and generation of reactive oxygen species are proposed mechanisms by which metabolic syndrome may increase the risk of dementia [17]. The formation of AGEs, which characterizes the hyperglycemia in type 2 diabetes mellitus (T2DM) and insulin resistance, may be accompanied by raised levels of neurotoxic methylglyoxal with high reactivity toward thiol groups, e.g., in microtubules in the neuronal cytoskeleton [55]. AGEs as well as ROS may act to enhance cerebral neuroinflammation. However, an early presumption that suppressing of neuroinflammation with non-steroid anti-inflammatory agents (NSAIDs) could arrest progressive precipitation of Aβ has now been abandoned, since NSAIDs such as ibuprofen or sulindac did not show therapeutic efficacy for AD treatment in clinical trials [56]. Nevertheless, the observed link between AD and the apolipoprotein E4 (ApoE4) allele also points to a role of dyslipidemia in the pathogenesis of AD. An early rough estimate indicated that having a single ApoE4 allele increases the AD risk 2- to 4-fold, whereas having two ApoE4 alleles increases the risk about 8- to 12-fold [57].

### 3.2. The Role of Glycation, AGE and RAGE in Alzheimer’s Disease

**Figure 1:**
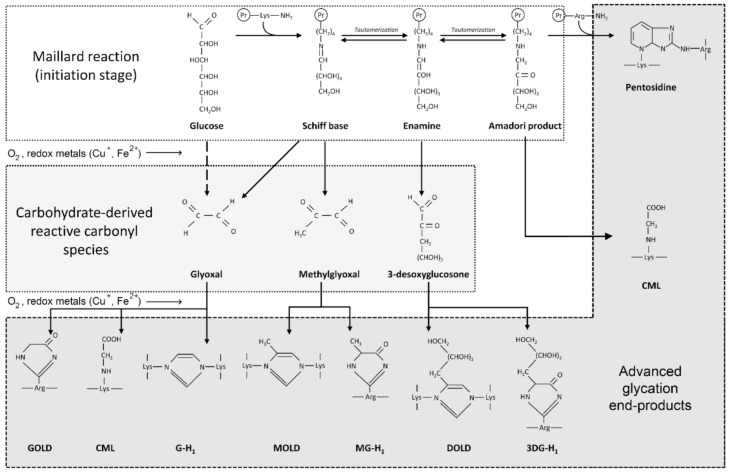
Schematic representation of the mechanisms of AGE formation through the Maillard reaction and reactive carbonyl mediated protein modification. Briefly, the reaction between the protein free amino group and carbonyl group of a reducing carbohydrate (glucose) results in formation of a Schiff base. The latter is subsequently rearranged to a more stable Amadori product that yields AGEs including carboxymethyllysine (CML) or pentosidine in a series of reactions. In addition, carbohydrate-derived reactive carbonyls that are formed from glucose and Schiff bases also interact with protein molecules resulting in formation of numerous AGEs including glyoxal-derived di-lysine imidazolium crosslink (GOLD), CML, glyoxal-derived hydroimidazolone (G-H1), methylglyoxal-derived di-lysine imidazolium crosslink (MOLD), methylglyoxal-derived hydroimidazolone (MG-H1), desoxyglucosone lysine dimer (DOLD) and 3-deoxyglucosone-derived hydroimidazolone 1 (3DG-H1). Formation of AGEs as well as carbohydrate-derived carbonyl species is stimulated by redox metals including copper and iron, which are involved in Fenton chemistry, as well as by oxidative stress.

High levels of glucose in diabetes mellitus, as well as monosaccharides such as fructose and glyceraldehyde, react non-enzymatically with sulfhydryl groups of proteins, lipids and nucleic acids, leading to the formation of advanced glycation end-products (AGEs). Briefly, protein glycation is a series of non-enzymatic reactions collectively termed the Maillard reaction [58] (Figure 1). At the first step, reaction between the protein free amino group and carbonyl group of a reducing carbohydrate (glucose) results in formation of a Schiff base that is subsequently rearranged to a more stable Amadori product. The latter undergoes a series of rearrangement, oxidation and dehydration reactions in the formation of AGEs. Another mechanism for AGE formation involves generation of reactive carbonyl species (glyoxal, methylglyoxal, 3-desoxyglucosone) that interact with amino acid residues of proteins resulting in AGE formation [59].

Irreversible modification of biological macromolecules during AGE formation results in alteration of its structure and function, and AGEs also possess toxic properties [60]. Glycation has been shown to induce aggregation of a wide spectrum of proteins including those implicated in the pathogenesis of AD [58]. A recent in vitro fluorescence study revealed a significant impact of glucose levels on Aβ1-40 aggregation, resulting in additional types of formed oligomers [61]. Correspondingly, Aβ glycation was associated with aggravation of amyloid neurotoxicity through up-regulation of RAGE and subsequent GSK-3 activation [62].

In addition, AGE accumulation may promote expression of amyloid precursor protein (APP) both in vivo and in vitro, whereas ROS scavenging was shown to inhibit this effect [63]. Further, it has been demonstrated that exposure to glycation metabolites, including methylglyoxal and glyceraldehyde, modifies Aβ42 structure, thus inducing its misfolding and accumulation [64]. AGE-RAGE axis activation in primary cortical neurons was shown to increase Aβ1-42 formation and tau phosphorylation through up-regulation of cathepsin B and asparagine endopeptidase (AEP) expression, respectively [65].

The role of AGEs in AD may be mediated by up-regulation of the receptor for advanced glycation end-products (RAGE). RAGE is a transmembrane protein of the immunoglobulin superfamily that in parallel with AGE interacts with multiple ligands including high-mobility group protein (B)1, S100 protein and Aβ, to name a few. Activated RAGE is involved in a variety of processes including inflammation, oxidative stress, apoptosis, autophagy, proliferation and migration [66]. Correspondingly, RAGE is considered to play a significant role in the pathogenesis of AD [67].

Aβ-RAGE interactions were also shown to induce BBB dysfunction through neuroinflammation- and oxidative-stress-dependent alteration of tight junctions [68]. Correspondingly, inhibition of RAGE in diabetic db/db mice resulted in a significant decrease in Aβ transport and neuronal apoptosis, and simultaneously an improved hippocampal neuroplasticity, altogether resulting in prevention of memory loss [69].

Moreover, RAGE was shown to be involved in infection-induced neuroinflammation with accompanying amyloid accumulation. Thus, in a cultured hCMEC/D3 cell line, *Porphyromonas gingivalis* infection significantly up-regulated RAGE expression, which was associated with Aβ influx into the cells [70]. Similar effects were observed in a model of Streptococcus-pneumoniae-induced meningitis [71].

As for the mechanisms of the role of RAGE in neuroinflammation, recent studies have demonstrated that RAGE activation may activate ERK1/2, JNK and p38 MAPK signaling, as well as ROS overproduction through NAPDH oxidase, with subsequent induction of the NF-kB pathway [72]. In turn, NF-kB activation was also shown to up-regulate RAGE expression, thus maintaining the vicious circle of neuroinflammation [73].

**Figure 2:**
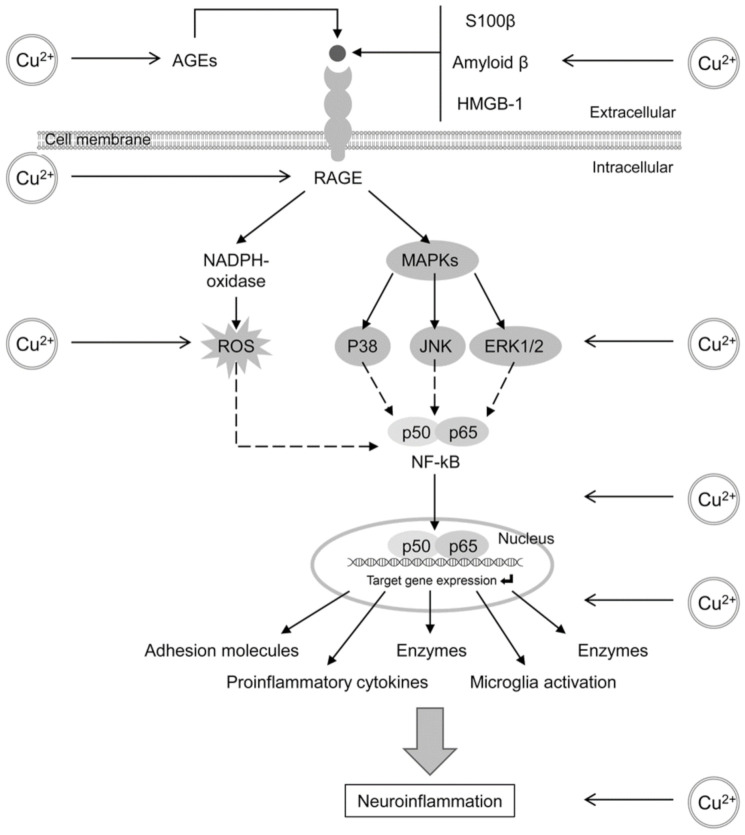
The role of AGE/RAGE signaling in neuroinflammation and its potential role as a mediator of neuroinflammatory effects of copper (Cu) and other metals. Binding of AGEs and other substrates (including Aβ, S100 protein and high-mobility group protein B1) to RAGE results in its activation with subsequent up-regulation of NADPH-oxidase and ROS generation, as well as MAPK (including p38, ERK1/2 and JNK) activation, which together cause NF-kB activation. The latter results in up-regulation of expression of target genes, including proinflammatory cytokines, adhesion molecules, chemokines, enzymes and subsequent microglial activation. Cu and potentially other redox metals (iron and manganese) may modulate the AGE/RAGE pathways. Specifically, Cu promotes AGE-formation and up-regulates the RAGE expression. Moreover, Cu exposure has been shown to increase Aβ aggregation, which may also act as a RAGE substrate. These AGE-dependent mechanisms may mediate the neuroinflammatory effects of Cu and other metals.

Consequently, both AGE production and RAGE signaling may be involved not only in promotion of A*β* production, but also in neuroinflammation (Figure 2), playing a significant role in neurotoxicity and neurodegeneration. Thus, the AGE/RAGE pathway may be considered a main mediator for the neurotoxic effects of various stressors.

## 4. The Role of Copper (Cu) and Iron (Fe) in Alzheimer’s Disease

### 4.1. General Aspects

Recent studies have demonstrated the involvement of metal overload and its subsequent neurotoxicity in the pathogenesis of Alzheimer’s disease [74], the most convincing data being obtained for Cu and Fe.

It is known that the presence in neurons of free ions of Cu and Fe will trigger deleterious Fenton-like reactions generating ROS and microinflammation [75]. Furthermore, it has been observed that the ceruloplasmin ferroxidase activity in blood is lower in subjects with MCI than in controls [76], while increased concentrations of circulating nonceruloplasmin Cu will increase the Cu transport into the brain [77]. A low ceruloplasmin activity may also result in increased Fe deposition in the brain, resulting in cognitive decline as seen in the rare disease aceruloplasminemia [78].

From animal experiments it is known that free Cu ions possess neurotoxic properties [79]. In 2003 Sparks and Schreurs reported that Cu excesses in drinking water together with increased cholesterol content in the chow for 10 weeks induced learning deficits in a rabbit model [80]. A community-based epidemiological study suggested that high dietary Cu in conjunction with a diet high in saturated fat led to cognitive decline [81]. Increased serum concentrations of nonceruloplasmin Cu appear to predict transition of mild cognitive impairment into AD [82]. However, observations in Wilson’s disease and aceruloplasminemia indicate that a cerebral elevation of either Cu or Fe concentration alone is not sufficient to initiate the Aβ-precipitates characterizing AD [78,83]. Interestingly, metabolic syndrome with NAFLD (nonalcoholic fatty liver disease) may lead to deranged hepatic synthesis of ceruloplasmin with altered blood levels of copper and iron [84].

The association of Fe with AD pathology is supported by the finding that high ferritin levels in cerebrospinal fluid (CSF) correlated with the transition of mild cognitive impairment (MCI) into AD, as observed in a cohort study [85]. Furthermore, CSF ferritin levels were strongly associated with cognitive decline in carriers of the ApoE4 allele [86]. Of interest is also the observation that the binding of Fe and Cu ions to phosphorylated tau protein precedes the formation of intracellular tangles [87]. It is also known that cations of these same transition elements accumulate in AD plaques enhancing the progression of the Aβ cascade [88]. 

Masaldan and colleagues have suggested that the complex pathological process of AD can be described as a ferroptosis [89], underscoring a role of iron interactions in addition to lipid dysmetabolism in the generation of the characteristic AD pathology.

Taken together, these data demonstrate that the redox metals Cu and Fe are involved in the complex AD pathogenesis. Given their role in oxidative stress, as well as the earlier discussed involvement in glycation processes and AGE/RAGE signaling in molecular mechanisms of AD, we propose that metals may play a significant role in AD etiology secondary to their impact on AGE generation and subsequent toxicity.

### 4.2. Glycation and AGE Toxicity in AD as Influenced by Cu

In the hypothesis of redox active “glycochelate” formation, Qian et al. [90] proposed that glycated proteins bind substantially higher numbers of catalytically active Fe and Cu atoms, thus promoting redox activity of such “chelates”, as assessed by increased ascorbic acid oxidation. Cu-catalyzed formation of AGEs in human serum albumin was shown to induce genotoxicity and inflammatory response through up-regulation of NF-kB, caspases 3 and 9, p53, cyclin D1 and p38-MAPK expression in cultured motor neuron cells [91]. It has also been proposed that albumin glycation may promote the Cu toxicity in AD [92].

Furthermore, it has been reported that the interaction between Cu and glycation may have a significant impact on the fate of Aβ. Specifically, Cu as well as Fe ions were shown to promote AGE-Aβ cross-linking and subsequent Aβ deposition [93]. The results of small-angle X-ray scattering analysis demonstrated that Cu cations interfere with AD Aβ peptide in a dose-dependent manner. At sub-equimolar concentrations Cu induces formation of elongated Aβ structures, whereas at higher levels the formation of Cu-induced Aβ1-42 ellipsoid oligomers is observed [94]. Apparently, Cu exposure can alter Aβ aggregation and promote its cross-linking with AGE [95].

It has clearly been shown that amyloid glycation interferes with Cu toxicity. Thus, Aβ1-40 glycation at Lys16 and Arg-5 results in superoxide formation that is known to interact with Cu^2+^ ions with subsequent generation of cytotoxic hydroxyl radicals via Fenton chemistry [79].

### 4.3. Glycation and AGE Toxicity in AD as Influenced by Fe

Several studies have addressed the role of iron in AGE formation, which may also underlie the potential involvement of Fe^2+^ in neurodegeneration. Specifically, the potential catalytic role of Fe^2+^ in the formation of AGEs in type 1 collagen has been clearly demonstrated [96]. In patients with β-thalassemia major, serum iron and especially non-transferrin-bound iron are typically elevated, and a positive correlation has been reported between this elevation and AGE (carboxymethyl-lysine and pentosidine) concentrations [97]. Correspondingly, in our recent experimental study Fe supplementation in obese rats induced a significant accumulation of AGEs and especially of CML in the liver [98]. Iron (Fe^3+^) is capable of inducing formation of DNA-AGE adducts, especially when combined with glyoxal and arginine [99]. However, direct evidence demonstrating the trilateral relationship between iron, AGE formation and Aβ accumulation and/or toxicity are lacking.

## 5. New Therapeutic Approaches to AD

In view of recent advantages in the understanding of AD pathogenesis and the role of AGEs as well as metal-induced glycation and neurodegeneration, new therapeutic strategies have been proposed addressing these pathogenetic targets.

### 5.1. Lipo-Glycemic Dysregulation—A Possible Therapeutic Target?

As molecular mechanisms in AD and insulin resistance seem related, it is tempting to hypothesize that drugs used for T2DM treatment could also be protective in AD. A phase II trial with rosiglitazone for 6 months reported improvements in memory in AD patients who did not possess the e4 allele of the ApoE gene, but a later phase III trial using the same drug failed to confirm a protective effect [30]. Another T2DM drug, metformin, was also reported to afford protection against memory loss [100], but a clinical trial did not confirm its alleged protective effect [101]. Dyslipidemia or hypercholesterolemia seem to increase the risk for dementia [102], an assumption that may be strengthened by the role of the ApoE4 allele as a predisposing factor for AD. Much research on ApoE in the CNS has focused on its critical role in shuttling cholesterol to neurons for the maintenance of cell membranes and synapses, and for their repair after injury [103]. However, statins appeared to have only a minor benefit, if any, in delaying AD progression [104].

In recent years, a new drug family has been the subject of growing interest, also in terms of protection against cognitive decline in AD, viz. the incretins. The two main endogenous incretins are GIP (the gastric inhibitory polypeptide) and GLP-1 (the glucagon-like peptide type 1). Animal studies have shown that some GLP-1 agonists could ameliorate neuroinflammation [105]. Of particular interest are the promising results obtained with the GLP-1 agonist liraglutide against cognitive decline, not only in rodent models [106], but also in a clinical double-blind trial [107]. At present, liraglutide is an approved drug both for T2DM and for obesity [108]. It is relevant here that the antioxidant resveratrol has also shown benefits for symptoms related to MetS and dementia [109]. Protection against ROS-promoted deteriorations of neuronal integrity is the mechanism suggested both for resveratrol and the incretins.

Another novel class of oral antidiabetics targeting incretins includes dipeptidyl peptidase IV inhibitors (gliptins). These agents are responsible for inhibition of incretin degradation thus promoting its half-life. Along with antidiabetic effects, gliptins possess significant protective effects against neurodegeneration [110]. Specifically, it has been demonstrated that dipeptidyl peptidase IV is up-regulated in AD brains and is colocalized with amyloid plaques [111]. In turn, long-term dipeptidyl peptidase IV inhibition by sitagliptin was shown to increase cerebral GLP-1 levels and reduce βAPP and Aβ accumulation, as well as neuroinflammation, in AD-prone rats [112].

### 5.2. Metal Chelation—A Rational Strategy?

**Figure 3:**
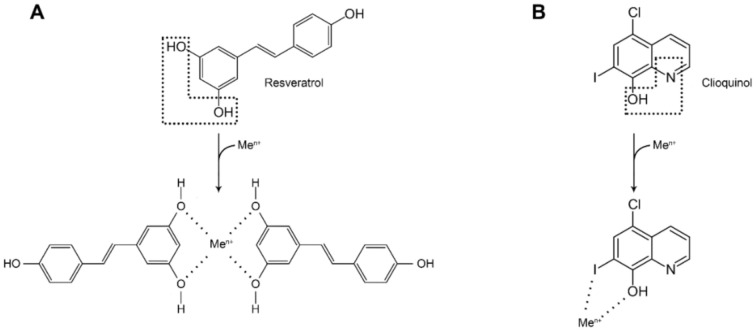
Resveratrol and clioquinol as metal-chelating agents. The proposed mechanisms of metal ion (Me^n+^) chelation by resveratrol (**A**) [104] and clioquinol (**B**) [108]. Dotted lines indicate functional groups responsible for metal chelation.

As discussed above, impaired copper and iron metabolism in AD brains may be accompanied by accelerated development of dementia. In accordance with this, an early study reported that iron chelation with deferoxamine (125 mg i.m. twice daily/5 days/week for 24 months) resulted in a significant reduction in the rate of decline of daily living skills in 48 AD patients, compared to AD patients receiving a placebo [113]. Another iron chelator, deferiprone has shown promising results in a mouse model [114]. However, only few metal chelating agents have been examined in clinical trials for the treatment of AD in recent years, viz. primarily clioquinol and PBT2 (5,7-dichloro-2-(dimethylamino)-methyl)-8-hydroxyquinoline). Promising observations have also been reported for another chelator, resveratrol [115,116] (Figure 3). The quinoline derivatives do chelate cerebral excesses of iron and copper in animal studies, and have been expected to retard the amyloid plaque progression in humans [117]. Although none of the studies on quinolines have actually shown clear clinical effects on AD progression, post-hoc analyses have claimed that the studies disclose a promising principle [118]. A limitation regarding clinical use of quinoline derivatives is that long-term use may give rise to serious side effects including mental health problems [119]. In addition, since the cognitive decline in AD seems to be driven also by a glycemic dysregulation in addition to a disturbed metal homeostasis, a monotherapy with metal chelation alone is not expected to reverse completely the pathological process.

### 5.3. Selenium Compounds as Protective Agents

**Figure 4:**
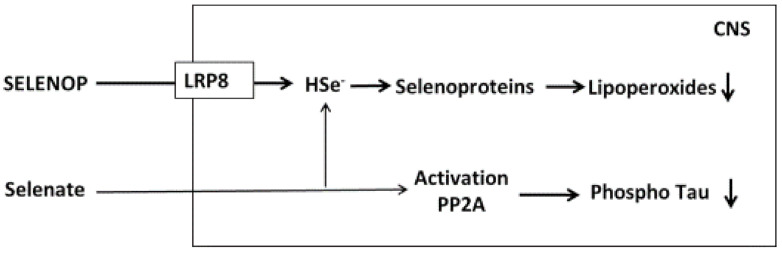
Uptake of selenoprotein P (SELENOP) and selenate in CNS. Selenoprotein P is imported into CNS via the ApoE-receptor LRP8 and is crucial for the synthesis of antioxidative selenoenzymes. Selenate is presumed to be imported by an anion carrier and is hypothesized to act as an activator of protein phosphatase 2 (PP2A), thereby reducing tau phosphorylation (see text). Selenate may also support selenoprotein synthesis.

Selenium is a trace element crucial to cerebral functions. During selenium depletion brain levels are maintained for a prolonged time at the expense of other tissues, whereas severe selenium deficiency causes irreversible brain damage [120]. The circulating selenium transporter, selenoprotein P (SELENOP), appears to have a special role in the delivery of selenium to the brain by entering neurons via the apolipoprotein E receptor 2 (LRP8), a member of the lipoprotein-receptor family that is expressed exclusively in the brain [120,121] (Figure 4). Interestingly, cholesterol and selenium are imported into neurons via this same receptor. While SELENOP is the important extracellular selenium transporter, the important intracellular antioxidants in neurons and glia are glutathione peroxidase 1 and 4 (GPX1 and GPX4) [122]. In addition, thioredoxin reductases are abundantly expressed in these cells. Selenium studies in animal models have given results that are in accordance with the observations from human surveys. Strikingly, high extracellular levels of selenoprotein P (SELENOP) have been found in the brains of rodents [123]. All regions of the mouse brain appear to be dependent on selenium for maintenance of proper functions [124]. Knock-out of the SEPP transporter in mice resulted in severe neurological dysfunction particularly when mice were fed a low selenium diet [72,125].

Evidence from human studies suggests a role for selenium and selenoproteins in protection against cognitive decline, especially in European countries where inhabitants are known to have low intakes of selenium in food. In the InCHIANTI cohort study of 1012 Italian participants aged 65 years or older, performance-based assessment scores of coordination were significantly reduced in participants with low plasma selenium compared to those with higher selenium [126]. In the French EVA cohort of 1166 people aged 60–70 years a significantly increased risk of cognitive decline was recorded over four years in participants with low plasma selenium at baseline [127]. In Spain, lower serum Se levels from elderly AD patients in comparison to MCI subjects have been reported [128]. A study from Brazil showed that daily supplementation with selenium-containing Brazil nuts over a period of 6 months, corresponding to about 250 µg Se/day, was associated with cognitive improvement as assessed by subtests of the CERAD panel [129]. Although the Preadvise study from USA with higher basal intake of selenium did not confirm a comparable improvement from supplementation [130], the conclusion in a recent meta-analysis was that sufficient evidence now exists for a reduced selenium status in AD brains as compared to the selenium status of healthy controls [131]. It is proposed that the existing inconsistencies regarding neuroprotective effects of Se in AD may occur due to differences in dietary Se intake worldwide, with better effects observed in Se-deficient populations. It is also relevant that selenium supplementation (200 µg/day) exerted a therapeutic improvement of glycemic control by inhibiting protein glycation and inflammatory response in a double-blind study on elderly Swedish subjects [132,133].

Existing experimental data have shown potential protective effects of physiological doses of selenium in AD pathogenesis. Specifically, Se treatment was found to reduce Aβ40 and Aβ42 production in SH-SY5Y cells and primary cultured rat cortical neurons [134]. Along with decreased APP levels, Se significantly increased the turnover of Aβ [135]. In addition, Se nanoparticles stabilized with chitosan were shown to inhibit metal-induced Aβ1-42 aggregation [136].

Another target for anti-amyloidogenic effects of Se may include inhibition of γ-secretase activity resulting in decreased Aβ1-40 production [137].

Sodium selenate supplementation has been found to deliver selenium to the brain [138] and to reduce tau phosphorylation [139]. In animal models, selenate appears to activate phosphatases and induce protective enzymes including glutathione peroxidases (GPXs), which may attenuate the intracellular burden of ROS. Early observations reporting selenate protection against cognitive decline [140] may be related to its protection of microtubules in the cytoskeleton. In accordance with this, Se has been shown reduce tau phosphorylation [141]. Inhibition of GSK-3β was also shown as the potential mechanism of Se-induced inhibition of tau accumulation and phosphorylation [142]. Similar effects were observed for the organic Se complex, Ebselen, which reduced tau phosphorylation at Thr231, Ser396 and Ser404 residues through modulation of PP2A and GSK-3β activity [143].

The reported inhibitory effects of Se on amyloidogenic pathways correspond to the observed Se-induced protection against Aβ toxicity. Specifically, co-exposure to Se significantly decreased Aβ cytotoxicity and ameliorated synaptic dysfunction [144], as well as memory, and neuropsychiatric impairments in AD mice [145].

One of the mechanisms underlying the potential protective effects of Se in Alzheimer’s disease may include inhibition of glycation and AGE formation with subsequent down-regulation of the AGE/RAGE pathway. Specifically, Se nanoparticles were shown to inhibit albumin glycation in a dose-dependent manner through their interaction with protein amino acid residues, together with ROS scavenging and inhibition of alpha-carbonyl formation [146].

It is also notable that Se may not only influence AGE formation, but may also modulate AGE signaling and toxicity. Particularly, the role of selenium-induced inhibition of AGE formation in prevention of p38 MAPK activation and subsequent COX-2 and P-selectin expression was demonstrated in human umbilical vein endothelial cells [147]. In streptozotocin-induced diabetic rats sodium selenite ameliorated hyperglycemia and insulin resistance, as well as down-regulated expression of RAGE and NF-kB [148].

Given the role of AGE in mediation of the interaction between Cu, Aβ-accumulation and neurotoxicity, it is proposed that Se may counteract Cu-induced neurodegeneration at least partially by influencing AGE formation. It is also proposed that metal coordination may play a significant role in the antioxidant effects of selenium. Specifically, selenoproteins containing selenium in the form of the amino acid selenocysteine, which confers a high affinity toward Cu(I) [149], will have potential to protect against copper toxicity. Correspondingly, selenocysteine and selenomethioneine were shown to reduce Cu/H_2_O_2_-mediated oxidative damage of DNA, and these effects of the Se compounds were independent of GPX activity, thus being indicative of the key role of Cu-Se coordination in the observed antioxidant activity [150]. In corroboration, a detailed study by Du et al. (2014) demonstrated that the His-rich SELENOP domain coordinates Cu^+^ and Cu^2+^ and prevents Cu-binding to Aβ42 as well as the subsequent aggregation and neurotoxicity [151]. Correspondingly, SELENOP was shown to reduce Cu^2+^-induced tau aggregation, as well as mitochondrial dysfunction and oxidative stress in cultured cortical neurons [152].

## 6. Conclusions

AD presents an increasing burden to society and a threat to human intellectual functions. The primary cause of this neurodegenerative disease has yet to be fully characterized. The amyloid cascade hypothesis has dominated the field for about 30 years, although new directions have been explored recently. Pharmacotherapeutic approaches built on knowledge about intracellular dysfunctions including the intracellular loading of amyloid before its extracellular translocation, which accompanies cell deterioration, may result in more efficient strategies.

The impact of glucose dysregulation with increased formation of advanced glycation end-products (AGEs) is underscored in the present review. The role of Cu and Fe in oxidative damage to structural macromolecules of the brain as well as in glucose and lipid dysmetabolism need to be addressed. Selenium compounds and incretins act as potent inhibitors of the pathological formation of AGEs, ROS, lipo-peroxides, amyloid and hyperphosphorylated tau proteins. Selenium compounds act as metal chelators and as intracellular protectors against derangement of microtubules and neuronal integrity. Optimized or pharmacological selenium intakes combined with incretin-targeting agents (e.g., GLP-agonists and dipeptidyl-peptidase IV inhibitors) in addition to glutaminyl cyclase inhibitors may be proposed as a possible treatment strategy for AD. Exposure routes for copper and iron in AD need further investigation.

## References

[1] Alzheimer’s Association (2021). 2021 Alzheimers disease facts and figures. Alzheimers Dement..

[2] Long J.M., Holtzman D.M. (2019). Alzheimer Disease: An Update on Pathobiology and Treatment Strategies. Cell.

[3] Sadigh-Eteghad S., Talebi M., Farhoudi M. (2012). Association of apolipoprotein E epsilon 4 allele with sporadic late onset Alzheimer’s disease. A meta-analysis. Neurosciences.

[4] Jacobs E.G., Kroenke C., Lin J., Epel E.S., Kenna H.A., Blackburn E.H., Rasgon N.L. (2013). Accelerated cell aging in female APOE-epsilon4 carriers: Implications for hormone therapy use. PLoS ONE.

[5] Liu Q., Zerbinatti C.V., Zhang J., Hoe H.S., Wang B., Cole S.L., Herz J., Muglia L., Bu G. (2007). Amyloid precursor protein regulates brain apolipoprotein E and cholesterol metabolism through lipoprotein receptor LRP1. Neuron.

[6] Schweizer U., Bohleber S., Zhao W., Fradejas-Villar N. (2021). The Neurobiology of Selenium: Looking Back and to the Future. Front. Neurosci.

[7] Deane R., Bell R.D., Sagare A., Zlokovic B.V. (2009). Clearance of amyloid-beta peptide across the blood-brain barrier: Implication for therapies in Alzheimer’s disease. CNS Neurol. Disord. Drug Targets.

[8] Bachmeier C., Paris D., Beaulieu-Abdelahad D., Mouzon B., Mullan M., Crawford F. (2013). A multifaceted role for apoE in the clearance of beta-amyloid across the blood-brain barrier. Neurodegener. Dis..

[9] Drachman D.A. (2014). The amyloid hypothesis, time to move on: Amyloid is the downstream result, not cause, of Alzheimer’s disease. Alzheimers Dement..

[10] Jahn H. (2013). Memory loss in Alzheimer’s disease. Dialogues Clin. Neurosci..

[11] Lim H.K., Jung W.S., Ahn K.J., Won W.Y., Hahn C., Lee S.Y., Kim I., Lee C.U. (2012). Relationships between hippocampal shape and cognitive performances in drug-naive patients with Alzheimer’s disease. Neurosci. Lett..

[12] Konishi K., Hori K., Tani M., Tomioka H., Kitajima Y., Akashi N., Inamoto A., Kurosawa K., Yuda H., Hanashi T. (2015). Hypothesis of Endogenous Anticholinergic Activity in Alzheimer’s Disease. Neurodegener. Dis..

[13] Wallace T.L., Bertrand D. (2013). Importance of the nicotinic acetylcholine receptor system in the prefrontal cortex. Biochem. Pharm..

[14] Parsons C.G., Stoffler A., Danysz W. (2007). Memantine: A NMDA receptor antagonist that improves memory by restoration of homeostasis in the glutamatergic system—Too little activation is bad, too much is even worse. Neuropharmacology.

[15] Surguchov A. (2020). Caveolin: A New Link Between Diabetes and AD. Cell Mol. Neurobiol..

[16] Pugazhenthi S., Qin L., Reddy P.H. (2017). Common neurodegenerative pathways in obesity, diabetes, and Alzheimer’s disease. Biochim. Biophys. Acta Mol. Basis Dis..

[17] Rojas-Gutierrez E., Munoz-Arenas G., Trevino S., Espinosa B., Chavez R., Rojas K., Flores G., Diaz A., Guevara J. (2017). Alzheimer’s disease and metabolic syndrome: A link from oxidative stress and inflammation to neurodegeneration. Synapse.

[18] Rani V., Deep G., Singh R.K., Palle K., Yadav U.C. (2016). Oxidative stress and metabolic disorders: Pathogenesis and therapeutic strategies. Life Sci..

[19] Prohaska J.R., Gybina A.A. (2004). Intracellular copper transport in mammals. J. Nutr..

[20] Muller U.C., Deller T., Korte M. (2017). Not just amyloid: Physiological functions of the amyloid precursor protein family. Nat. Rev. Neurosci..

[21] Mockett B.G., Richter M., Abraham W.C., Muller U.C. (2017). Therapeutic Potential of Secreted Amyloid Precursor Protein APPsalpha. Front. Mol. Neurosci..

[22] Guo Q., Wang Z., Li H., Wiese M., Zheng H. (2012). APP physiological and pathophysiological functions: Insights from animal models. Cell Res..

[23] Richter M.C., Ludewig S., Winschel A., Abel T., Bold C., Salzburger L.R., Klein S., Han K., Weyer S.W., Fritz A.K. (2018). Distinct in vivo roles of secreted APP ectodomain variants APPsalpha and APPsbeta in regulation of spine density, synaptic plasticity, and cognition. EMBO J..

[24] Morley J.E., Farr S.A., Nguyen A.D., Xu F. (2019). Editorial: What is the Physiological Function of Amyloid-Beta Protein?. J. Nutr. Health Aging.

[25] Kent S.A., Spires-Jones T.L., Durrant C.S. (2020). The physiological roles of tau and Abeta: Implications for Alzheimer’s disease pathology and therapeutics. Acta Neuropathol..

[26] Williams T.L., Serpell L.C. (2011). Membrane and surface interactions of Alzheimer’s Abeta peptide—Insights into the mechanism of cytotoxicity. FEBS J..

[27] Warmlander S., Tiiman A., Abelein A., Luo J., Jarvet J., Soderberg K.L., Danielsson J., Graslund A. (2013). Biophysical studies of the amyloid beta-peptide: Interactions with metal ions and small molecules. ChemBioChem.

[28] Gilman S., Koller M., Black R.S., Jenkins L., Griffith S.G., Fox N.C., Eisner L., Kirby L., Rovira M.B., Forette F. (2005). Clinical effects of Abeta immunization (AN1792) in patients with AD in an interrupted trial. Neurology.

[29] Panza F., Solfrizzi V., Imbimbo B.P., Tortelli R., Santamato A., Logroscino G. (2014). Amyloid-based immunotherapy for Alzheimer’s disease in the time of prevention trials: The way forward. Expert Rev. Clin. Immunol..

[30] Gold M. (2017). Phase II clinical trials of anti-amyloid beta antibodies: When is enough, enough?. Alzheimers Dement. N. Y..

[31] Perez-Garmendia R., Gevorkian G. (2013). Pyroglutamate-Modified Amyloid Beta Peptides: Emerging Targets for Alzheimer’s Disease Immunotherapy. Curr. Neuropharmacol..

[32] Andreeva T.V., Lukiw W.J., Rogaev E.I. (2017). Biological Basis for Amyloidogenesis in Alzheimer’s Disease. Biochemistry.

[33] Ghosal K., Vogt D.L., Liang M., Shen Y., Lamb B.T., Pimplikar S.W. (2009). Alzheimer’s disease-like pathological features in transgenic mice expressing the APP intracellular domain. Proc. Natl. Acad. Sci. USA.

[34] Sakono M., Zako T. (2010). Amyloid oligomers: Formation and toxicity of Abeta oligomers. FEBS J..

[35] Alsunusi S., Kumosani T.A., Glabe C.G., Huwait E.A., Moselhy S.S. (2020). In vitro study of the mechanism of intraneuronal beta-amyloid aggregation in Alzheimer’s disease. Arch. Physiol. Biochem..

[36] Shang F., Taylor A. (2011). Ubiquitin-proteasome pathway and cellular responses to oxidative stress. Free Radic. Biol. Med..

[37] Nassif N.D., Cambray S.E., Kraut D.A. (2014). Slipping up: Partial substrate degradation by ATP-dependent proteases. IUBMB Life.

[38] Saez I., Vilchez D. (2014). The Mechanistic Links Between Proteasome Activity, Aging and Age-related Diseases. Curr. Genom..

[39] Keck S., Nitsch R., Grune T., Ullrich O. (2003). Proteasome inhibition by paired helical filament-tau in brains of patients with Alzheimer’s disease. J. Neurochem..

[40] Dickey C.A., Koren J., Zhang Y.J., Xu Y.F., Jinwal U.K., Birnbaum M.J., Monks B., Sun M., Cheng J.Q., Patterson C. (2008). Akt and CHIP coregulate tau degradation through coordinated interactions. Proc. Natl. Acad. Sci. USA.

[41] Tseng B.P., Green K.N., Chan J.L., Blurton-Jones M., LaFerla F.M. (2008). Abeta inhibits the proteasome and enhances amyloid and tau accumulation. Neurobiol. Aging.

[42] Bjorklund G., Aaseth J., Dadar M., Chirumbolo S. (2019). Molecular Targets in Alzheimer’s Disease. Mol. Neurobiol..

[43] Reeg S., Grune T. (2015). Protein Oxidation in Aging: Does It Play a Role in Aging Progression?. Antioxid. Redox Signal..

[44] Aaseth J., Alexander J., Bjorklund G., Hestad K., Dusek P., Roos P.M., Alehagen U. (2016). Treatment strategies in Alzheimer’s disease: A review with focus on selenium supplementation. Biometals.

[45] Andreyev A.Y., Kushnareva Y.E., Murphy A.N., Starkov A.A. (2015). Mitochondrial ROS Metabolism: 10 Years Later. Biochemistry.

[46] Nixon R.A., Wegiel J., Kumar A., Yu W.H., Peterhoff C., Cataldo A., Cuervo A.M. (2005). Extensive involvement of autophagy in Alzheimer disease: An immuno-electron microscopy study. J. Neuropathol. Exp. Neurol..

[47] Nilsson P., Loganathan K., Sekiguchi M., Matsuba Y., Hui K., Tsubuki S., Tanaka M., Iwata N., Saito T., Saido T.C. (2013). Abeta secretion and plaque formation depend on autophagy. Cell Rep..

[48] Leal N.S., Martins L.M. (2021). Mind the Gap: Mitochondria and the Endoplasmic Reticulum in Neurodegenerative Diseases. Biomedicines.

[49] Fang E.F. (2019). Mitophagy and NAD^+^ inhibit Alzheimer disease. Autophagy.

[50] Wang J.Z., Liu F. (2008). Microtubule-associated protein tau in development, degeneration and protection of neurons. Prog. Neurobiol..

[51] Aaseth J., Buha A., Wallace D.R., Bjorklund G. (2020). Xenobiotics, Trace Metals and Genetics in the Pathogenesis of Tauopathies. Int. J. Environ. Res. Public Health.

[52] Shukla V., Skuntz S., Pant H.C. (2012). Deregulated Cdk5 activity is involved in inducing Alzheimer’s disease. Arch. Med. Res..

[53] Mushtaq G., Greig N.H., Anwar F., Al-Abbasi F.A., Zamzami M.A., Al-Talhi H.A., Kamal M.A. (2016). Neuroprotective Mechanisms Mediated by CDK5 Inhibition. Curr. Pharm. Des..

[54] Kivipelto M., Mangialasche F., Ngandu T. (2018). Lifestyle interventions to prevent cognitive impairment, dementia and Alzheimer disease. Nat. Rev. Neurol..

[55] Schalkwijk C.G., Stehouwer C.D.A. (2020). Methylglyoxal, a Highly Reactive Dicarbonyl Compound, in Diabetes, Its Vascular Complications, and Other Age-Related Diseases. Physiol. Rev..

[56] Grossberg G.T., Tong G., Burke A.D., Tariot P.N. (2019). Present Algorithms and Future Treatments for Alzheimer’s Disease. J. Alzheimers Dis..

[57] Farrer L.A., Cupples L.A., Haines J.L., Hyman B., Kukull W.A., Mayeux R., Myers R.H., Pericak-Vance M.A., Risch N., van Duijn C.M. (1997). Effects of age, sex, and ethnicity on the association between apolipoprotein E genotype and Alzheimer disease. A meta-analysis. APOE and Alzheimer Disease Meta Analysis Consortium. JAMA.

[58] Chaudhuri J., Bains Y., Guha S., Kahn A., Hall D., Bose N., Gugliucci A., Kapahi P. (2018). The Role of Advanced Glycation End Products in Aging and Metabolic Diseases: Bridging Association and Causality. Cell Metab..

[59] Brings S., Fleming T., Freichel M., Muckenthaler M.U., Herzig S., Nawroth P.P. (2017). Dicarbonyls and Advanced Glycation End-Products in the Development of Diabetic Complications and Targets for Intervention. Int. J. Mol. Sci..

[60] Rungratanawanich W., Qu Y., Wang X., Essa M.M., Song B.J. (2021). Advanced glycation end products (AGEs) and other adducts in aging-related diseases and alcohol-mediated tissue injury. Exp. Mol. Med..

[61] Alghamdi A., Forbes S., Birch D.J.S., Vyshemirsky V., Rolinski O.J. (2021). Detecting beta-amyloid glycation by intrinsic fluorescence—Understanding the link between diabetes and Alzheimer’s disease. Arch. Biochem. Biophys..

[62] Li X.H., Du L.L., Cheng X.S., Jiang X., Zhang Y., Lv B.L., Liu R., Wang J.Z., Zhou X.W. (2013). Glycation exacerbates the neuronal toxicity of beta-amyloid. Cell Death Dis..

[63] Ko S.Y., Lin Y.P., Lin Y.S., Chang S.S. (2010). Advanced glycation end products enhance amyloid precursor protein expression by inducing reactive oxygen species. Free Radic. Biol. Med..

[64] Fawver J.N., Schall H.E., Petrofes Chapa R.D., Zhu X., Murray I.V. (2012). Amyloid-beta metabolite sensing: Biochemical linking of glycation modification and misfolding. J. Alzheimer’s Dis..

[65] Batkulwar K., Godbole R., Banarjee R., Kassaar O., Williams R.J., Kulkarni M.J. (2018). Advanced Glycation End Products Modulate Amyloidogenic APP Processing and Tau Phosphorylation: A Mechanistic Link between Glycation and the Development of Alzheimer’s Disease. ACS Chem. Neurosci..

[66] Lee E.J., Park J.H. (2013). Receptor for Advanced Glycation Endproducts (RAGE), Its Ligands, and Soluble RAGE: Potential Biomarkers for Diagnosis and Therapeutic Targets for Human Renal Diseases. Genom. Inf..

[67] Cai Z., Liu N., Wang C., Qin B., Zhou Y., Xiao M., Chang L., Yan L.J., Zhao B. (2016). Role of RAGE in Alzheimer’s Disease. Cell Mol. Neurobiol..

[68] Wan W., Chen H., Li Y. (2014). The potential mechanisms of Abeta-receptor for advanced glycation end-products interaction disrupting tight junctions of the blood-brain barrier in Alzheimer’s disease. Int. J. Neurosci..

[69] Wang H., Chen F., Du Y.F., Long Y., Reed M.N., Hu M., Suppiramaniam V., Hong H., Tang S.S. (2018). Targeted inhibition of RAGE reduces amyloid-beta influx across the blood-brain barrier and improves cognitive deficits in db/db mice. Neuropharmacology.

[70] Zeng F., Liu Y., Huang W., Qing H., Kadowaki T., Kashiwazaki H., Ni J., Wu Z. (2020). Receptor for advanced glycation end products up-regulation in cerebral endothelial cells mediates cerebrovascular-related amyloid beta accumulation after Porphyromonas gingivalis infection. J. Neurochem..

[71] Barichello T., Generoso J.S., Giridharan V.V., Collodel A., Dominguini D., Petronilho F., Dal-Pizzol F. (2020). Receptor for advanced glycation end products mediates meningitis-triggered amyloid-β accumulation and cognitive impairment. Alzheimers Dement..

[72] Ray R., Juranek J.K., Rai V. (2016). RAGE axis in neuroinflammation, neurodegeneration and its emerging role in the pathogenesis of amyotrophic lateral sclerosis. Neurosci. Biobehav. Rev..

[73] Tobon-Velasco J.C., Cuevas E., Torres-Ramos M.A. (2014). Receptor for AGEs (RAGE) as mediator of NF-kB pathway activation in neuroinflammation and oxidative stress. CNS Neurol. Disord. Drug Targets.

[74] Bush A.I. (2003). Copper, zinc, and the metallobiology of Alzheimer disease. Alzheimer Dis. Assoc. Disord..

[75] Ward R.J., Dexter D.T., Crichton R.R. (2015). Neurodegenerative diseases and therapeutic strategies using iron chelators. J. Trace Elem. Med. Biol..

[76] Torsdottir G., Kristinsson J., Snaedal J., Johannesson T. (2011). Ceruloplasmin and iron proteins in the serum of patients with Alzheimer’s disease. Dement. Geriatr. Cogn. Dis. Extra.

[77] Squitti R., Ghidoni R., Simonelli I., Ivanova I.D., Colabufo N.A., Zuin M., Benussi L., Binetti G., Cassetta E., Rongioletti M. (2018). Copper dyshomeostasis in Wilson disease and Alzheimer’s disease as shown by serum and urine copper indicators. J. Trace Elem. Med. Biol..

[78] Kono S. (2013). Aceruloplasminemia: An update. Int. Rev. Neurobiol..

[79] Fica-Contreras S.M., Shuster S.O., Durfee N.D., Bowe G.J.K., Henning N.J., Hill S.A., Vrla G.D., Stillman D.R., Suralik K.M., Sandwick R.K. (2017). Glycation of Lys-16 and Arg-5 in amyloid-beta and the presence of Cu^2+^ play a major role in the oxidative stress mechanism of Alzheimer’s disease. J. Biol. Inorg. Chem..

[80] Sparks D.L., Schreurs B.G. (2003). Trace amounts of copper in water induce beta-amyloid plaques and learning deficits in a rabbit model of Alzheimer’s disease. Proc. Natl. Acad. Sci. USA.

[81] Morris M.C., Evans D.A., Tangney C.C., Bienias J.L., Schneider J.A., Wilson R.S., Scherr P.A. (2006). Dietary copper and high saturated and trans fat intakes associated with cognitive decline. Arch. Neurol..

[82] Squitti R., Ghidoni R., Siotto M., Ventriglia M., Benussi L., Paterlini A., Magri M., Binetti G., Cassetta E., Caprara D. (2014). Value of serum nonceruloplasmin copper for prediction of mild cognitive impairment conversion to Alzheimer disease. Ann. Neurol..

[83] Meenakshi-Sundaram S., Mahadevan A., Taly A.B., Arunodaya G.R., Swamy H.S., Shankar S.K. (2008). Wilson’s disease: A clinico-neuropathological autopsy study. J. Clin. Neurosci..

[84] Aigner E., Theurl I., Haufe H., Seifert M., Hohla F., Scharinger L., Stickel F., Mourlane F., Weiss G., Datz C. (2008). Copper availability contributes to iron perturbations in human nonalcoholic fatty liver disease. Gastroenterology.

[85] Ayton S., Faux N.G., Bush A.I., Alzheimer’s Disease Neuroimaging Initiative (2015). Ferritin levels in the cerebrospinal fluid predict Alzheimer’s disease outcomes and are regulated by APOE. Nat. Commun..

[86] Ayton S., Faux N.G., Bush A.I. (2017). Association of Cerebrospinal Fluid Ferritin Level with Preclinical Cognitive Decline in APOE-epsilon4 Carriers. JAMA Neurol..

[87] Ahmadi S., Zhu S., Sharma R., Wilson D.J., Kraatz H.B. (2019). Interaction of metal ions with tau protein. The case for a metal-mediated tau aggregation. J. Inorg. Biochem..

[88] Bourassa M.W., Leskovjan A.C., Tappero R.V., Farquhar E.R., Colton C.A., Van Nostrand W.E., Miller L.M. (2013). Elevated copper in the amyloid plaques and iron in the cortex are observed in mouse models of Alzheimer’s disease that exhibit neurodegeneration. Biomed. Spectrosc. Imaging.

[89] Masaldan S., Bush A.I., Devos D., Rolland A.S., Moreau C. (2019). Striking while the iron is hot: Iron metabolism and ferroptosis in neurodegeneration. Free Radic. Biol. Med..

[90] Qian M., Liu M., Eaton J.W. (1998). Transition metals bind to glycated proteins forming redox active “glycochelates”: Implications for the pathogenesis of certain diabetic complications. Biochem. Biophys. Res. Commun..

[91] Marques C.M.S., Nunes E.A., Lago L., Pedron C.N., Manieri T.M., Sato R.H., Oliveira V.X.J., Cerchiaro G. (2017). Generation of Advanced Glycation End-Products (AGEs) by glycoxidation mediated by copper and ROS in a human serum albumin (HSA) model peptide: Reaction mechanism and damage in motor neuron cells. Mutat. Res..

[92] Siotto M., Squitti R. (2018). Copper imbalance in Alzheimer’s disease: Overview of the exchangeable copper component in plasma and the intriguing role albumin plays. Coord. Chem. Rev..

[93] Loske C., Gerdemann A., Schepl W., Wycislo M., Schinzel R., Palm D., Riederer P., Munch G. (2000). Transition metal-mediated glycoxidation accelerates cross-linking of beta-amyloid peptide. Eur J. Biochem..

[94] Ryan T.M., Kirby N., Mertens H.D., Roberts B., Barnham K.J., Cappai R., Pham Cle L., Masters C.L., Curtain C.C. (2015). Small angle X-ray scattering analysis of Cu^2+^-induced oligomers of the Alzheimer’s amyloid beta peptide. Metallomics.

[95] Rahmadi A., Steiner N., Munch G. (2011). Advanced glycation endproducts as gerontotoxins and biomarkers for carbonyl-based degenerative processes in Alzheimer’s disease. Clin. Chem. Lab. Med..

[96] Xiao H., Cai G., Liu M. (2007). Fe^2+^-catalyzed non-enzymatic glycosylation alters collagen conformation during AGE-collagen formation in vitro. Arch. Biochem. Biophys..

[97] Mirlohi M.S., Yaghooti H., Shirali S., Aminasnafi A., Olapour S. (2018). Increased levels of advanced glycation end products positively correlate with iron overload and oxidative stress markers in patients with beta-thalassemia major. Ann. Hematol..

[98] Chen S.H., Yuan K.C., Lee Y.C., Shih C.K., Tseng S.H., Tinkov A.A., Skalny A.V., Chang J.S. (2020). Iron and Advanced Glycation End Products: Emerging Role of Iron in Androgen Deficiency in Obesity. Antioxidants.

[99] Shahab U., Tabrez S., Khan M.S., Akhter F., Khan M.S., Saeed M., Ahmad K., Srivastava A.K., Ahmad S. (2014). Immunogenicity of DNA-advanced glycation end product fashioned through glyoxal and arginine in the presence of Fe^3+^: Its potential role in prompt recognition of diabetes mellitus auto-antibodies. Chem. Biol. Interact..

[100] Alagiakrishnan K., Sankaralingam S., Ghosh M., Mereu L., Senior P. (2013). Antidiabetic drugs and their potential role in treating mild cognitive impairment and Alzheimer’s disease. Discov. Med..

[101] Kuan Y.C., Huang K.W., Lin C.L., Hu C.J., Kao C.H. (2017). Effects of metformin exposure on neurodegenerative diseases in elderly patients with type 2 diabetes mellitus. Prog. Neuropsychopharmacol. Biol. Psychiatry.

[102] Feinkohl I., Janke J., Hadzidiakos D., Slooter A., Winterer G., Spies C., Pischon T. (2019). Associations of the metabolic syndrome and its components with cognitive impairment in older adults. BMC Geriatr..

[103] Lane-Donovan C., Herz J. (2014). Is apolipoprotein e required for cognitive function in humans? Implications for Alzheimer drug development. JAMA Neurol..

[104] Davis K.A.S., Bishara D., Perera G., Molokhia M., Rajendran L., Stewart R.J. (2020). Benefits and Harms of Statins in People with Dementia: A Systematic Review and Meta-Analysis. J. Am. Geriatr. Soc..

[105] Qin L., Chong T., Rodriguez R., Pugazhenthi S. (2016). Glucagon-Like Peptide-1-Mediated Modulation of Inflammatory Pathways in the Diabetic Brain: Relevance to Alzheimer’s Disease. Curr. Alzheimer Res..

[106] Wicinski M., Socha M., Malinowski B., Wodkiewicz E., Walczak M., Gorski K., Slupski M., Pawlak-Osinska K. (2019). Liraglutide and its Neuroprotective Properties-Focus on Possible Biochemical Mechanisms in Alzheimer’s Disease and Cerebral Ischemic Events. Int. J. Mol. Sci..

[107] Watson K.T., Wroolie T.E., Tong G., Foland-Ross L.C., Frangou S., Singh M., McIntyre R.S., Roat-Shumway S., Myoraku A., Reiss A.L. (2019). Neural correlates of liraglutide effects in persons at risk for Alzheimer’s disease. Behav. Brain Res..

[108] Suliman M., Buckley A., Al Tikriti A., Tan T., le Roux C.W., Lessan N., Barakat M. (2019). Routine clinical use of liraglutide 3 mg for the treatment of obesity: Outcomes in non-surgical and bariatric surgery patients. Diabetes Obes. Metab..

[109] Diaz-Gerevini G.T., Repossi G., Dain A., Tarres M.C., Das U.N., Eynard A.R. (2016). Beneficial action of resveratrol: How and why?. Nutrition.

[110] Juillerat-Jeanneret L. (2014). Dipeptidyl peptidase IV and its inhibitors: Therapeutics for type 2 diabetes and what else?. J. Med. Chem..

[111] Bernstein H.G., Dobrowolny H., Keilhoff G., Steiner J. (2018). Dipeptidyl peptidase IV, which probably plays important roles in Alzheimer disease (AD) pathology, is upregulated in AD brain neurons and associates with amyloid plaques. Neurochem. Int..

[112] D’Amico M., Di Filippo C., Marfella R., Abbatecola A.M., Ferraraccio F., Rossi F., Paolisso G. (2010). Long-term inhibition of dipeptidyl peptidase-4 in Alzheimer’s prone mice. Exp. Gerontol..

[113] McLachlan D.R., Smith W.L., Kruck T.P. (1993). Desferrioxamine and Alzheimer’s disease: Video home behavior assessment of clinical course and measures of brain aluminum. Ther. Drug Monit..

[114] Rao S.S., Portbury S.D., Lago L., Bush A.I., Adlard P.A. (2020). The Iron Chelator Deferiprone Improves the Phenotype in a Mouse Model of Tauopathy. J. Alzheimer’s Dis..

[115] Grinan-Ferre C., Bellver-Sanchis A., Izquierdo V., Corpas R., Roig-Soriano J., Chillon M., Andres-Lacueva C., Somogyvari M., Soti C., Sanfeliu C. (2021). The pleiotropic neuroprotective effects of resveratrol in cognitive decline and Alzheimer’s disease pathology: From antioxidant to epigenetic therapy. Ageing Res. Rev..

[116] Gülçin İ. (2010). Antioxidant properties of resveratrol: A structure-activity insight. Innov. Food Sci. Emerg. Technol..

[117] Bush A.I. (2002). Metal complexing agents as therapies for Alzheimer’s disease. Neurobiol. Aging.

[118] Faux N.G., Ritchie C.W., Gunn A., Rembach A., Tsatsanis A., Bedo J., Harrison J., Lannfelt L., Blennow K., Zetterberg H. (2010). PBT2 rapidly improves cognition in Alzheimer’s Disease: Additional phase II analyses. J. Alzheimer’s Dis..

[119] Galatti L., Giustini S.E., Sessa A., Polimeni G., Salvo F., Spina E., Caputi A.P. (2005). Neuropsychiatric reactions to drugs: An analysis of spontaneous reports from general practitioners in Italy. Pharm. Res..

[120] Burk R.F., Hill K.E. (2009). Selenoprotein P-expression, functions, and roles in mammals. Biochim. Biophys. Acta.

[121] Schweizer U., Brauer A.U., Kohrle J., Nitsch R., Savaskan N.E. (2004). Selenium and brain function: A poorly recognized liaison. Brain Res. Brain Res. Rev..

[122] Mitozo P.A., de Souza L.F., Loch-Neckel G., Flesch S., Maris A.F., Figueiredo C.P., Dos Santos A.R., Farina M., Dafre A.L. (2011). A study of the relative importance of the peroxiredoxin-, catalase-, and glutathione-dependent systems in neural peroxide metabolism. Free Radic. Biol. Med..

[123] Steinbrenner H., Sies H. (2013). Selenium homeostasis and antioxidant selenoproteins in brain: Implications for disorders in the central nervous system. Arch. Biochem. Biophys..

[124] Nakayama A., Hill K.E., Austin L.M., Motley A.K., Burk R.F. (2007). All regions of mouse brain are dependent on selenoprotein P for maintenance of selenium. J. Nutr..

[125] Caito S.W., Milatovic D., Hill K.E., Aschner M., Burk R.F., Valentine W.M. (2011). Progression of neurodegeneration and morphologic changes in the brains of juvenile mice with selenoprotein P deleted. Brain Res..

[126] Shahar A., Patel K.V., Semba R.D., Bandinelli S., Shahar D.R., Ferrucci L., Guralnik J.M. (2010). Plasma selenium is positively related to performance in neurological tasks assessing coordination and motor speed. Mov. Disord..

[127] Berr C., Balansard B., Arnaud J., Roussel A.M., Alperovitch A. (2000). Cognitive decline is associated with systemic oxidative stress: The EVA study. Etude du Vieillissement Arteriel. J. Am. Geriatr. Soc..

[128] Gonzalez-Dominguez R., Garcia-Barrera T., Gomez-Ariza J.L. (2014). Homeostasis of metals in the progression of Alzheimer’s disease. Biometals.

[129] Cardoso B.R., Roberts B.R., Bush A.I., Hare D.J. (2015). Selenium, selenoproteins and neurodegenerative diseases. Metallomics.

[130] Kryscio R.J., Abner E.L., Caban-Holt A., Lovell M., Goodman P., Darke A.K., Yee M., Crowley J., Schmitt F.A. (2017). Association of Antioxidant Supplement Use and Dementia in the Prevention of Alzheimer’s Disease by Vitamin E and Selenium Trial (PREADViSE). JAMA Neurol..

[131] Varikasuvu S.R., Prasad V.S., Kothapalli J., Manne M. (2019). Brain Selenium in Alzheimer’s Disease (BRAIN SEAD Study): A Systematic Review and Meta-Analysis. Biol. Trace Elem. Res..

[132] Alehagen U., Aaseth J., Alexander J., Johansson P., Larsson A. (2020). Supplemental selenium and coenzyme Q10 reduce glycation along with cardiovascular mortality in an elderly population with low selenium status—A four-year, prospective, randomised, double-blind placebo-controlled trial. J. Trace Elem. Med. Biol..

[133] Alehagen U., Alexander J., Aaseth J., Larsson A. (2019). Decrease in inflammatory biomarker concentration by intervention with selenium and coenzyme Q10: A subanalysis of osteopontin, osteoprotergerin, TNFr1, TNFr2 and TWEAK. J. Inflamm..

[134] Gwon A.R., Park J.S., Park J.H., Baik S.H., Jeong H.Y., Hyun D.H., Park K.W., Jo D.G. (2010). Selenium attenuates A beta production and A beta-induced neuronal death. Neurosci. Lett..

[135] Song G.L., Chen C., Wu Q.Y., Zhang Z.H., Zheng R., Chen Y., Jia S.Z., Ni J.Z. (2018). Selenium-enriched yeast inhibited beta-amyloid production and modulated autophagy in a triple transgenic mouse model of Alzheimer’s disease. Metallomics.

[136] Vicente-Zurdo D., Romero-Sanchez I., Rosales-Conrado N., Leon-Gonzalez M.E., Madrid Y. (2020). Ability of selenium species to inhibit metal-induced Abeta aggregation involved in the development of Alzheimer’s disease. Anal. Bioanal. Chem..

[137] Li G.Z., Liu F., Xu C., Li J.Y., Xu Y.J. (2018). Selenium and Zinc against Abeta25-35-Induced Cytotoxicity and Tau Phosphorylation in PC12 Cells and Inhibits gamma-cleavage of APP. Biol. Trace Elem. Res..

[138] Cardoso B.R., Roberts B.R., Malpas C.B., Vivash L., Genc S., Saling M.M., Desmond P., Steward C., Hicks R.J., Callahan J. (2019). Supranutritional Sodium Selenate Supplementation Delivers Selenium to the Central Nervous System: Results from a Randomized Controlled Pilot Trial in Alzheimer’s Disease. Neurotherapeutics.

[139] Tan X.L., Wright D.K., Liu S., Hovens C., O’Brien T.J., Shultz S.R. (2016). Sodium selenate, a protein phosphatase 2A activator, mitigates hyperphosphorylated tau and improves repeated mild traumatic brain injury outcomes. Neuropharmacology.

[140] Tolonen M., Halme M., Sarna S. (1985). Vitamin E and selenium supplementation in geriatric patients: A double-blind preliminary clinical trial. Biol. Trace Elem. Res..

[141] Zhang Z.H., Wen L., Wu Q.Y., Chen C., Zheng R., Liu Q., Ni J.Z., Song G.L. (2017). Long-Term Dietary Supplementation with Selenium-Enriched Yeast Improves Cognitive Impairment, Reverses Synaptic Deficits, and Mitigates Tau Pathology in a Triple Transgenic Mouse Model of Alzheimer’s Disease. J. Agric. Food Chem..

[142] van Eersel J., Ke Y.D., Liu X., Delerue F., Kril J.J., Gotz J., Ittner L.M. (2010). Sodium selenate mitigates tau pathology, neurodegeneration, and functional deficits in Alzheimer’s disease models. Proc. Natl. Acad. Sci. USA.

[143] Xie Y., Tan Y., Zheng Y., Du X., Liu Q. (2017). Ebselen ameliorates beta-amyloid pathology, tau pathology, and cognitive impairment in triple-transgenic Alzheimer’s disease mice. J. Biol. Inorg. Chem..

[144] Godoi G.L., de Oliveira Porciuncula L., Schulz J.F., Kaufmann F.N., da Rocha J.B., de Souza D.O., Ghisleni G., de Almeida H.L. (2013). Selenium compounds prevent amyloid beta-peptide neurotoxicity in rat primary hippocampal neurons. Neurochem. Res..

[145] Van der Jeugd A., Parra-Damas A., Baeta-Corral R., Soto-Faguas C.M., Ahmed T., LaFerla F.M., Gimenez-Llort L., D’Hooge R., Saura C.A. (2018). Reversal of memory and neuropsychiatric symptoms and reduced tau pathology by selenium in 3xTg-AD mice. Sci. Rep..

[146] Yu S., Zhang W., Liu W., Zhu W., Guo R., Wang Y., Zhang D., Wang J. (2015). The inhibitory effect of selenium nanoparticles on protein glycation in vitro. Nanotechnology.

[147] Li Y.B., Han J.Y., Jiang W., Wang J. (2011). Selenium inhibits high glucose-induced cyclooxygenase-2 and P-selectin expression in vascular endothelial cells. Mol. Biol. Rep..

[148] Pillai S.S., Sugathan J.K., Indira M. (2012). Selenium downregulates RAGE and NFkappaB expression in diabetic rats. Biol. Trace Elem. Res..

[149] Zimmerman M.T., Bayse C.A., Ramoutar R.R., Brumaghim J.L. (2015). Sulfur and selenium antioxidants: Challenging radical scavenging mechanisms and developing structure-activity relationships based on metal binding. J. Inorg. Biochem..

[150] Battin E.E., Perron N.R., Brumaghim J.L. (2006). The central role of metal coordination in selenium antioxidant activity. Inorg. Chem..

[151] Du X., Wang Z., Zheng Y., Li H., Ni J., Liu Q. (2014). Inhibitory effect of selenoprotein P on Cu^+^/Cu^2+^-induced Abeta42 aggregation and toxicity. Inorg. Chem..

[152] Du X., Zheng Y., Wang Z., Chen Y., Zhou R., Song G., Ni J., Liu Q. (2014). Inhibitory act of selenoprotein P on Cu^+^/Cu^2+^-induced tau aggregation and neurotoxicity. Inorg. Chem..

